# Fertility preservation using controlled ovarian stimulation in breast cancer: a comparative study of neoadjuvant and adjuvant settings

**DOI:** 10.1007/s10815-026-03812-6

**Published:** 2026-02-20

**Authors:** Moran Shapira, Chen Berkovitz, Myriam Safrai, Jigal Haas, Tal Sella, Adva Aizer, Dror Meirow, Raoul Orvieto

**Affiliations:** 1 IVF Institute, Sheba Medical Center, Ramat Gan, Israel; 2 Fertility Preservation Center, Obstetrics & Gynecology Department, Sheba Medical Center, Ramat Gan, Israel; 3 Tel Aviv University, Tel Aviv, Israel; 4 Department of Oncology, Sheba Medical Center, Ramat Gan, Israel

**Keywords:** Fertility preservation, Breast cancer, Neoadjuvant chemotherapy, Random-start

## Abstract

**Purpose:**

To compare the time to initiation of first therapeutic treatment and the outcomes of controlled ovarian stimulation (COS) in breast cancer patients undergoing fertility preservation (FP) in the neoadjuvant chemotherapy (NAC) versus adjuvant chemotherapy (AC) treatment settings.

**Methods:**

A retrospective cohort study involving patients with stage 1–3 breast cancer treated with NAC/AC, who underwent FP with random-start COS, between 2015–2023. Baseline, oncologic and COS characteristics and outcomes were collected. Time points related to cancer diagnosis, FP, and first oncologic intervention (surgery or chemotherapy) were calculated.

**Results:**

70 NAC-treated patients were compared to 42 AC-treated patients. Groups were similar in terms of age, marital status, parity and BMI. NAC-treated patients had more advanced disease, more often with lymph node involvement. Median time from diagnosis to FP consult (17 vs 30 days) and to stimulation start (22 vs 59 days) was significantly shorter for NAC-treated patients. Median time from diagnosis to first therapeutic intervention was slightly longer for NAC-treated patients (45 vs 41 days, *p* < 0.05). Stimulation characteristics were comparable apart from a less frequent use of hCG trigger among NAC-treated patients (4% vs 16%, *p* < 0.05). First stimulation cycle outcomes such as peak E2 levels, number of retrieved oocytes and fertilization rate were similar, though median number of M2 oocytes was higher in NAC- treated patients (11 vs 8, *p* < 0.05).

**Conclusion:**

Expedited patient care at the neoadjuvant setting led to a statistically significant, yet clinically minimal, delay of cancer therapy. Neither oncologic treatment timelines nor FP outcomes were compromised in the NAC setting.

## Introduction

Breast cancer is the most common malignancy diagnosed in individuals of reproductive age [1, 2]. In this population, the disease more often exhibits biologically aggressive features [3, 4], making chemotherapy a key component of treatment [5]. Beyond the gonadotoxic effects of chemotherapy, the need to postpone pregnancy due to prolonged adjuvant therapies may further impact future fertility [6]. Given that many young breast cancer patients have not yet completed childbearing at the time of diagnosis, FP- typically involving COS followed by oocyte or embryo cryopreservation- is often considered [6, 7].

Since oocyte or embryo cryopreservation must occur before the initiation of chemotherapy [8], the urgency of FP depends on the treatment approach. In cases of adjuvant chemotherapy, fertility preservation is typically performed after the initial treatment (surgery), during the natural interval that occurs as part of the postoperative recovery period before the initiation of chemotherapy. As such, fertility preservation does not dictate the timeline of oncologic treatment. In contrast, in the neoadjuvant chemotherapy setting, fertility preservation must take place before the initial treatment (chemotherapy), meaning that, by necessity, it determines the timing of the oncologic treatment.

In recent years, the use of neoadjuvant systemic therapy has increased [9, 10]. Neoadjuvant therapy increases the likelihood of breast-conserving surgery over mastectomy and allows for histologic evaluation of tumor response to systemic therapy, providing valuable prognostic insights. However, concerns regarding potential delays in systemic therapy, or the risk of inadequate FP outcomes due to time limitations, may discourage both oncologists and patients from pursuing FP, particularly in the setting of neoadjuvant systemic therapy [11–13].

In this study, we aimed to assess whether the time from cancer diagnosis to the first therapeutic intervention in patients pursuing FP is influenced by the type of chemotherapy (neoadjuvant vs. adjuvant), as well as whether FP outcomes differ when performed prior to neoadjuvant chemotherapy compared to adjuvant chemotherapy.

## Methods

### Study population

This retrospective study included reproductive-aged breast cancer patients who underwent FP at a tertiary medical between 2015 and 2023. All participants had initiated COS for the purpose of oocyte or embryo cryopreservation shortly after their cancer diagnosis and prior to the initiation of chemotherapy. Patients who did not receive chemotherapy or who had missing data were excluded. Exposure groups were defined by the timing of systemic therapy—those who underwent COS prior to receiving neoadjuvant chemotherapy (NAC group), and those who underwent COS prior to adjuvant chemotherapy (AC group).

### Fertility preservation consultation and COS protocol

Typically, during the study period, all reproductive-aged breast cancer patients were offered a fertility preservation consultation upon diagnosis. The consultation was conducted by a reproductive endocrinology and infertility specialist with expertise in oncologic fertility preservation. Consideration was given to marital status, family-building goals, and planned treatment. National health insurance provided financial coverage for COS in patients with up to one child. Patients scheduled for neoadjuvant chemotherapy were generally encouraged to initiate COS with minimal delay, whereas those scheduled for adjuvant chemotherapy were allowed a broader time window. Antagonist-based protocols were used, and random-start stimulation was performed as a standard, to avoid delay in cancer therapy. Gonadotrophins were administrated in variable doses, depending on patient’s age, BMI, and previous response to stimulation on repeat cycles. Gonadotropin doses were further adjusted according to serum E2 levels and vaginal ultrasound measurement obtained every 2–3 days.

In patients with hormone-receptor positive breast cancer cotreatment with Tamoxifen was applied. Tamoxifen (20 mg) was added once a rise of E2 levels was detected and antagonist injections commenced. Final follicular maturation was induced when the leading follicle measured > 17 mm, with either hCG (Ovitrelle 250 mcg, Merck), GnRH- agonist (GnRH-a) (Decapeptyl 0.2 mg) or the dual trigger (Ovitrelle 250 mcg and Decapeptyl 0.2 mg). A transvaginal, ultrasound-guided follicular aspiration was conducted 36 h after triggering. The number of cycle attempts for each patient was determined through a collaborative decision-making process involving the patient, the fertility specialist, and the treating oncologist, with consideration given to the treatment schedule and the outcomes of the initial COS cycle.

### Data collection

Institutional ethics committee approval was obtained for the study (SMC 9383–22). Electronic medical records and pathology reports were reviewed to extract demographic data, germline *BRCA1/2* mutation status, tumor grade, estrogen and progesterone receptor-status, human epidermal growth factor receptor (HER-2/neu) status, ki67 score, lymph node involvement and breast cancer stage. In patients treated with adjuvant chemotherapy, tumor size and lymph node status were extracted from pathology reports of surgical specimens. In patients treated with neoadjuvant chemotherapy, tumor size and lymph node status were defined based on pre-treatment clinical evaluation.

The following stimulation characteristics and outcomes were extracted for the first stimulation cycle: timing of stimulation initiation (early follicular, late follicular, luteal), initial gonadotropin dose, total gonadotropins dose, number of stimulation days, estrogen co-treatment, type of ovulation trigger, peak E2 levels, number of retrieved oocytes, number of mature (MII) oocytes, fertilization rate. The following data were collected for the entire FP process: number of stimulation cycles per patient, cumulative number of oocytes cryopreserved, and cumulative number of embryos cryopreserved.

Several time intervals were calculated and compared between the groups. Time to FP consult was defined as the interval from the date of diagnosis (the date of first positive biopsy) to first FP consult. Time to COS was defined as the interval from the date of diagnosis to initiation of COS. Time to first therapeutic intervention was defined as the interval between the date of diagnosis and the initiation of chemotherapy in the NAC group or surgical treatment in the AC group. For both study groups, time to first therapeutic intervention was also compared with that of a historical cohort of 152 reproductive-aged breast cancer patients who did not undergo fertility preservation [14].

### Statistical analysis

Statistical analysis was conducted with the use of SciPy, version 1.0.0. Normality of distribution was evaluated with the use of histograms and Q-Q plots. Continuous variables are presented as mean ± SD or median (IQR) as appropriate. Categoric variables are presented as *n* (%). Normally distributed continuous variables were compared by means of Student *t* test, and nonnormally distributed numbers were compared by means of Mann–Whitney test. Categoric variables were compared by means of chi-square. A *P* value of < 0.05 was considered statistically significant. A univariate linear regression model was used to assess the individual impact of each potential confounder on 1 st stimulation cycle outcomes (number of MII oocytes). Covariates with a *p*-value < 0.1 in the univariate analysis were included in the final multivariate linear regression model assessing first stimulation outcomes.

## Results

Between 2015 and 2023, 138 women with newly diagnosed early breast cancer underwent COS for FP. Of these, 19 did not receive chemotherapy and 6 had missing data, resulting in a final study cohort of 113 patients with 70 undergoing embryo or oocyte cryopreservation prior to neoadjuvant chemotherapy (NAC group), and 43 prior to adjuvant chemotherapy (AC group). Patient and disease characteristics are summarized in Table 1. NAC and AC groups were comparable in terms of age, body mass index (BMI), marital status, nulliparity rate, and prevalence of germline BRCA1/2 mutations. Patients treated with NAC were characterized by more aggressive disease phenotype, as evidenced by a trend toward higher tumor grade (grade 3, 73.8% vs 55.8%, *p* < 0.05), a significantly higher rate of lymph node involvement (61.4% vs 30.2%, *p* < 0.05), and a more advanced stage (stage III, 28.5% vs 9.3%, *p* < 0.05).

**Table 1:** Baseline and tumor characteristics

*P*-value	NAC group (*n* = 70)	AC group (*n* = 43)	
0.43	32.37 ± 4.48	33.07 ± 4.82	**Age**, years (mean ± SD)
Marital status			
0.39	32 (45.7)	21 (48.8)	Single, *n* (%)
	35 (50)	22 (51.2)	Married, *n* (%)
	3 (4.3)	0 (0)	Divorced, *n* (%)
0.58	37 (52.9)	18 (41.9)	**Nulliparity, *n*** **(%)**
0.58	22.74 ± 3.21	22.29 ± 3.13	**BMI**, kg/m^2^ (mean ± SD)
0.55	25 (41)	17 (50)	**BRCA positive (%)**
0.06	31 (44.3)	27 (62.8)	**Hormone receptor positive** ***n*, (%)**
< 0.05	26 (37.1)	5 (11.6)	**Her 2 positive,** ***n*** **(%)**
Ki67			
0.22	2 (2.9)	3 (7.1)	1, *n* (%)
	11 (16.2)	11 (26.2)	2, *n* (%)
	55 (80.9)	3 (7.1)	3, *n* (%)
Grade			
0.06	0 (0)	0 (0)	1, *n* (%)
	16 (26.2)	19 (44.2)	2,* n* (%)
	45 (73.8)	24 (55.8)	3, *n* (%)
< 0.05	43 (61.4)	13 (30.2)	**Axillary lymph nodes involvement,** ***n*** **(%)**
Stage			
< 0.05	3 (4.3)	19 (44.2)	I, *n* (%)
	47 (67.1)	20 (46.5)	II, *n* (%)
	20 (28.5)	4 (9.3)	III, *n* (%)

Baseline and tumor features of breast cancer patients who underwent fertility preservation prior to neoadjuvant (NAC) or adjuvant (AC) chemotherapy. Values are presented as mean ± SD or *n* (%). *NAC* neoadjuvant chemotherapy; *AC* adjuvant chemotherapy, *BMI* body mass index, *BRCA* breast cancer gene, *HER2* human epidermal growth factor receptor 2

In the AC group, 36 patients (83.72%) received FP following surgery, while the remaining 7 patients, initiated FP prior to undergoing surgery. Median time to first FP consult (17 days [IQR 13–28] 30 days [IQR 18–61], *p* < 0.05) and time to COS (22 days [IQR16-33.25] vs 59 [IQR 39–84], *p* < 0.05) were significantly shorter for the NAC group. Nonetheless, time to first therapeutic intervention was significantly longer for the NAC group (45 days [IQR 35.75–57.25] vs 41 [IQR 27–53], *p* < 0.05). Time to first therapeutic intervention was further evaluated using a historical cohort of young breast cancer patients who did not undergo fertility preservation [14]. In the neoadjuvant setting, the median time to first therapeutic intervention among patients not undergoing fertility preservation was significantly shorter (41.4 days [IQR 29.79–51.54], *p* < 0.05) compared with patients who underwent fertility preservation (45 days [IQR 35.75–57.25]). In the adjuvant setting, the median time to first therapeutic intervention did not differ significantly between patients who did (41 days [IQR 27–53]) and did not undergo fertility preservation (45 days [IQR 29–64], *p* = 0.16).

Characteristics and outcomes of the first stimulation cycle are presented in Table 2. The timing of stimulation initiation, duration of stimulation, initial gonadotropin dose, and co-treatment with Tamoxifen were comparable between the groups. A trend toward a higher cumulative gonadotropin dose was observed in the NAC group (3200 [IQR 2350- 4050] vs 2950 [IQR 2100- 3512], *p* = 0.09). HCG trigger was more often used in the AC group (4% vs 16%, *p* < 0.05), while GnRH-a and Dual trigger were more often used in the NAC group (96% vs 84%, *p* < 0.05). NAC and AC groups did not differ significantly in median peak estradiol levels and number of retrieved oocytes (14 [IQR 8.75–24] vs 13 [IQR 6–18], *p* = 0.11). However, median number of MII oocytes was significantly higher in the NAC group (11 [IQR 5.5–18]) compared to the AC group (8 [IQR 8–12.5], *p* = 0.024). Fertilization rates did not differ significantly between the groups.

**Table 2:** First controlled ovarian stimulation characteristics and outcomes by chemotherapy setting

*P*-value	NAC group (*n* = 70)	AC group (*n* = 43)	
Stimulation start timing			
0.274	42 (60)	31 (72.1)	Early follicular, *n* (%)
	6 (8.6)	1 (2.3)	Late follicular, *n* (%)
	22 (31.4)	11 (25.6)	Luteal, *n* (%)
0.421	10 [9, 12]	10 [9, 11]	**Stimulation days** (median [Q1, Q3])
0.497	300 [225, 350]	300 [225, 350]	**Gonadotropins initial dose**, IU (median [Q1, Q3])
0.090	3200 [2350, 4050]	2950 [2100, 3512]	**Gonadotropins total dose**, IU (median [Q1, Q3])
0.978	36 (51.4)	22 (51.2)	**Tamoxifen/letrozole co-treatment,** ***n*** **(%)**
Ovulation Trigger			
< 0.05	3 (4)	7 (16)	hCG, *n* (%)
	67 (96)	36 (84)	GnRH-a/Dual, *n* (%)
0.19	48,617 [5119, 14674]	6828 [4192, 11524]	**Maximal E2 levels**, pmol/L (median [Q1, Q3])
0.11	14 [8.75, 24]	13 [6, 18]	**Retrieved oocytes** (median [Q1, Q3])
< 0.05	11 [5.5, 18]	8 [4, 12.5]	**MII oocytes** (median [Q1, Q3])
0.20	76.43 ± 16.59	70.1 ± 20.91	**Fertilization rate, %** (mean ± SD)

First-cycle ovarian stimulation characteristics and outcomes for patients undergoing fertility preservation prior to neoadjuvant (NAC) or adjuvant (AC) chemotherapy. *COS* controlled ovarian stimulation, *GnRH-a* gonadotropin-releasing hormone agonist, *hCG* human chorionic gonadotropin, *MII* metaphase II oocytes, *E2* estradiol, *IU* international units

As shown in Table 3, univariate linear regression analysis was conducted to evaluate the effect of potential confounders on the number of MII oocytes. Only chemotherapy setting and ovulation trigger were significantly associated with MII oocyte number: NAC was linked to a greater number of MII oocytes, while hCG trigger was associated with fewer. After adjusting for trigger type in a multivariate regression analysis, the association between chemotherapy type and MII oocyte count became non-significant.

**Table 3:** Predictors of mature oocyte yield: linear regression models

	Univariate Analysis			Multivariate Analysis		
	β	SE	*P*	β	SE	*P*
NAC vs AC	3.65	1.75	0.04	2.84	1.75	0.11
Cumulative dose	0.0004	0.001	0.49			
Trigger (hCG vs GnRH-a/Dual)	−7.27	2.78	0.01	−6.33	2.81	0.03
Her 2 (positive vs negative)	−1.2370	2.059	0.549			
Hormone receptors (positive vs negative)	1.4722	1.756	1.756			
Grade (3 vs 2)	0.1319	1.841	0.943			
Stage (3 vs 2/1)	−0.4710	2.209	0.832			
Lymph node (positive vs negative)	2.4375	1.743	0.165			

Univariate and multivariate linear regression analyses evaluating predictors of the number of mature (MII) oocytes retrieved during the first controlled ovarian stimulation cycle. *β* regression coefficient, *SE* standard error, *NAC* neoadjuvant chemotherapy, *AC* adjuvant chemotherapy, *GnRH-a* gonadotropin-releasing hormone agonist

Prior to initiating chemotherapy, 6 patients (8.6%) in the NAC group underwent more than one cycle, compared to 18 patients (41.9%) in the AC group (*p* < 0.01). After inclusion of repeat cycles, the median cumulative number of MII oocytes was similar between the NAC and AC groups, with 11 (IQR 5–15) and 10.5 (IQR 5–20.25), respectively. Of the total cohort, 48 patients chose embryo storage (41.86% in the AC group vs 42.86% in the NAC group), 44 opted for oocyte storage (44.19% in the AC group vs 35.71% in the NAC group), and 16 elected to store both embryos and oocytes (11.62% in the AC group vs 15.71% in the NAC group), *p* = 0.763. In five cases, COS was attempted but did not result in the storage of any gametes or embryos. When comparing the groups, by the conclusion of the FP process, NAC and AC patients who chose only oocyte cryopreservation stored a similar number of eggs (13.5 [IQR 8.75–21.75] vs. 12 [IQR 7.25–15.5], *p* = 0.15). Similarly, NAC and AC patients who chose only embryo cryopreservation stored a similar number of embryos (5 [IQR 2.5–8.5] vs. 6 [IQR 4–9], *p* = 0.41).

## Discussion

The intersection of cancer treatment and FP presents both emotional and clinical challenges. While patients understandably prioritize survival, it is essential that oncologists and fertility specialists convey that parenthood remains a viable option which doesn’t necessarily compromise oncologic outcome [15, 16]. Beyond offering the possibility of future motherhood, FP can also provide a significant psychological benefit, serving as a source of hope and empowerment [17]. For breast cancer patients scheduled to receive adjuvant chemotherapy, integrating FP into the treatment timeline is generally straightforward. FP is typically performed after the initial therapeutic intervention- surgery- and therefore does not affect the timing of that intervention or delay the overall oncologic treatment. However, for patients undergoing NAC, the scenario is more complex, as the timing and extent of FP may ultimately postpone the start of first therapeutic treatment.

In this study of 130 young breast cancer patients undergoing COS prior to chemotherapy, the time to first therapeutic intervention was only slightly longer in the neoadjuvant setting. Chemotherapy was initiated after a median of 45 days after diagnosis in the neoadjuvant group, whereas surgery was performed after a median of 41 days from diagnosis in those treated with adjuvant chemotherapy. Both medians fall within the recommended period for initiating the cancer therapy after diagnosis (6–8 weeks) [18]. More specifically, young women receiving adjuvant therapy who experience surgical delays beyond six weeks have been shown to have shorter survival [19], while in the neoadjuvant setting, delays exceeding 60 days from diagnosis are associated with decreased survival [20]. Thus, at the upper end of the interquartile range and beyond, some women might have benefited from a shorter interval from diagnosis to first treatment. At the same time, given that omitting fertility preservation would, at most, only minimally shorten this interval, it cannot be concluded that fertility preservation itself caused any meaningful delay in initiating therapy in either study group. It is important to note that for patients with triple-negative tumours, even shorter delays of approximately 42 days may negatively affect prognosis [21]. For these high-risk patients, timely initiation of therapy remains highly critical. Larger studies are needed to further clarify the potential impact of fertility preservation–related delays on the initiation of chemotherapy in this population.

Previous studies have primarily examined treatment timelines in the context of neoadjuvant chemotherapy, reporting a time lapse of approximately 40 days between cancer diagnosis and the initiation of NAC when fertility preservation is incorporated [14, 22–24] a finding consistent with our results. The initiation of neoadjuvant chemotherapy after a median of 45 days in the current study was made possible through expedited management, as reflected by significantly shorter intervals from diagnosis to FP consultation and stimulation. In comparison, patients receiving adjuvant chemotherapy reached first intervention (surgery) within a similar timeframe, but without the need for such an accelerated coordination, since chemotherapy followed surgery rather than preceding it. The avoidance of delays in initiating stimulation was further facilitated by the liberal use of the random-start stimulation protocol, which allowed for the immediate initiation of controlled ovarian stimulation (COS), regardless of the timing within the menstrual cycle. This approach potentially reduced the overall FP timeline by 2–4 weeks, while maintaining oocyte retrieval numbers, mature oocyte yield, and fertilization rates [25, 26]. Our findings are consistent with previous studies, which also demonstrate that using the random-start protocol for FP does not cause delays in planned oncologic treatment [23, 24].

It is noteworthy that most patients in the AC group, initiated COS only after surgery, thereby missing the opportunity to utilize the time available between diagnosis and surgery for fertility preservation. Notably, time to first fertility preservation referral and time to initiation of ovarian stimulation were significantly longer in the adjuvant setting. This likely reflects a more permissive institutional approach in the adjuvant setting, where referrals for fertility consultation often occur later, after surgery, unlike in the neoadjuvant setting, where patients are typically referred soon after diagnosis. This approach is facilitated by the fact that chemotherapy should be initiated up to 6–8 weeks after surgery [18], providing a sufficient window to complete at least one to two cycles of fertility preservation before systemic treatment begins, without compromising the timing of therapy.

Indeed, more than half of the AC group were able to complete more than one stimulation cycle, in contrast to the majority of NAC patients, who underwent only a single cycle. Despite this, both groups ultimately stored a similar cumulative number of oocytes or embryos by the end of the FP process. This outcome may be attributed to the first stimulation cycle, during which the NAC group yielded a significantly higher number of mature (MII) oocytes (11 vs. 8, *p* = 0.024), despite a comparable number of retrieved oocytes. Although some studies have reported that positive hormone receptor status [27] and lower tumor grade [28] are associated with improved COS outcomes, the differing oncologic characteristics of the NAC groups could not explain the observed difference in mature oocyte yield. As shown in Table 3, MII oocyte yield in the current study was not associated with hormone receptor status or any measure of tumor burden, including cancer grade, stage, or lymph node involvement. The higher MII yield in the NAC group may be simply explained by the type of final oocyte maturation trigger. The NAC group more frequently received a GnRH agonist or dual trigger. Among the possible advantages of GnRH agonist for ovulation triggering is the endogenous induction of both an LH and FSH surge, more closely mimicking the physiologic mid-cycle gonadotropin profile than hCG alone, which provides only an LH-like stimulus. The FSH surge is thought to enhance cumulus expansion, oocyte detachment, and nuclear maturation, thereby increasing the proportion of mature oocytes retrieved [29–31]. Indeed, in a univariate linear regression model of the current cohort, GnRH agonist or dual trigger use was positively associated with MII oocyte number. As shown in Table 3, adjusting for trigger type in multivariate analysis rendered the association between chemotherapy type and MII oocyte number non-significant.

Our study expands current knowledge by offering a broader perspective on both oncologic and reproductive aspects of FP prior to NAC. By adjusting for oncologic and stimulation-related variables, we were able to effectively demonstrate the non-inferiority of FP in the neoadjuvant setting, while also highlighting the feasibility of meeting established oncologic quality standards. Nonetheless, several limitations should be acknowledged. Most notably, the retrospective design of the study poses inherent limitations. Additionally, because our data were drawn from an IVF clinic database, we were only able to include patients who ultimately elected to undergo FP, thus excluding potentially valuable information about those who chose not to pursue FP. Another limitation is related to calculation and comparison of time intervals, most notably time to first therapeutic intervention. Given that histopathological confirmation by biopsy represents the definitive diagnostic step before systemic therapy, and in line with common practice in the literature, this date was used as the anchor time point for timeline calculations. This time point may have been indirectly influenced by the timing of earlier evaluation processes, such as the initial abnormal clinical breast examination or breast imaging. In addition, both external and internal referral processes may have affected timelines but could not be accounted for in the current study. Finally, long-term reproductive outcomes, including utilization of stored gametes or embryos and post-treatment pregnancy rates, were not assessed. Currently, most available data pertains to the use of cryopreserved embryos. A recent meta-analysis reported an 18% utilization rate, a clinical pregnancy rate of 50%, and a live birth rate of 33% [32].

In conclusion, this study supports the feasibility of COS for FP prior to NAC, specifically with the use of random start COS with GnRH-a or Dural trigger. We could demonstrate that neither treatment timelines nor fertility FP outcomes are compromised compared to the more flexible context of performing FP in the adjuvant setting. The decision to pursue FP should not be determined solely by the timing of planned chemotherapy. Rather, timely coordination between oncology and fertility teams is key to facilitating appropriate and effective care.

## Data Availability

The data that support the findings of this study are not openly available due to reasons of sensitivity and are available from the corresponding author upon reasonable request.
